# Construct development: The Suicide Trigger Scale (STS-2), a measure of a hypothesized suicide trigger state

**DOI:** 10.1186/1471-244X-10-110

**Published:** 2010-12-14

**Authors:** Zimri Yaseen, Curren Katz, Matthew S Johnson, Daniel Eisenberg, Lisa J Cohen, Igor I Galynker

**Affiliations:** 1 Beth Israel Medical Center, New York, New York, USA; 2 Teachers College, Columbia University, New York, New York, USA; 3 National Institute of Mental Health, Bethesda, Maryland, USA

## Abstract

**Background:**

This study aims to develop the construct of a 'suicide trigger state' by exploring data gathered with a novel psychometric self-report instrument, the STS-2.

**Methods:**

The STS-2, was administered to 141 adult psychiatric patients with suicidal ideation. Multiple statistical methods were used to explore construct validity and structure.

**Results:**

Cronbach's alpha (0.949) demonstrated excellent internal consistency. Factor analyses yielded two-component solutions with good agreement. The first component described near-psychotic somatization and ruminative flooding, while the second described frantic hopelessness. ROC analysis determined an optimal cut score for a history of suicide attempt, with significance of p < 0.03. Logistic regression analysis found items sensitive to history of suicide attempt described ruminative flooding, doom, hopelessness, entrapment and dread.

**Conclusions:**

The STS-2 appears to measure a distinct and novel clinical entity, which we speculatively term the 'suicide trigger state.' High scores on the STS-2 associate with reported history of past suicide attempt.

## Background

Though many chronic factors placing an individual at increased risk for suicide are well established, the acute factors that lead a person to make a suicide attempt (SA) are not known. Chronic risk factors include suicidal ideation (SI), history of suicide attempts, severe psychopathology, history of psychiatric hospitalization, substance abuse, and poor social supports[1,2]. Among these, SI and history of previous SA are most prominent and most relied upon in general clinical practice[3-7].

At present, however, no instruments are well established for the prediction of imminent SA [7]. Moreover, current measures of suicidality, including the Suicide Assessment Scale,[8-10] Suicide Intent Scale,[11,12] and Motto and Bostrom's proposed scale,[13] rely heavily on self-report of overt suicidal thoughts and plans. However, acutely suicidal individuals often deny or hide their suicidal intent,[14,15] and the presence of a plan for suicide is a poor predictor of attempt, as many attempters report only fleeting ideation and no premeditated plan[4]. In fact, a recent study reported an average interval of only 10 minutes between the onset of SI and the actual suicidal act[16].

Past research suggests that transition from SI to SA may be triggered by specific affective, behavioral, and cognitive factors [17-19]. However, the exact nature of these "trigger" factors or whether they constitute a distinct "trigger state" is not known. Esposito et al.,[17] reported that in adolescents, after controlling for depression, only anger and affect dysregulation differentiated multiple from single suicide attempters. Nock and Kazdin[18] have identified negative automatic thinking as a risk factor for suicide attempts. This type of cognition might be related to the "diffuse ruminative thought process"[20] characteristic of psychosis. Indeed, Radomsky et al.,[21] showed that 30.2% of patients with psychosis make a suicidal attempt at some point in their life.

Furthermore, although controversial, a growing body of evidence links panic attacks to suicidal behavior in patients with depression [22,23]. It has been reported that this link persists even when controlling for depression, substance abuse and sociodemographic characteristics[22,23]. Weissman et al.,[24] found that 20% of subjects with panic disorder and 12% of those with panic attacks had made suicide attempts.

Finally, Schnyder et al.,[25] observed that panic and self-report of "loss of control" seems to be a distinct state that occurs before individuals attempt suicide, while Busch et al., [15] found in an acute psychological autopsy study of 76 completed inpatient suicides, that nearly 80 percent both denied suicidal ideation in the days before their suicides and, using items from the Schedule for Affective Disorders and Schizophrenia (SADS), met criteria for severe to extreme anxiety or agitation, and Hendin et.al., [26] identified acute high affective intensity, in particular desperation, as the distinguishing feature of suicide completers in a case controlled psychological autopsy study.

In the course of our work on psychotic panic,[27] we have encountered a distinct psychopathologic state or syndrome related to panic and psychosis,[27,23] fitting with the findings of Hendin, Busch, and Snyder described above, which is reported by many suicide attempters as occurring immediately prior to their suicide attempt. In accordance with the aforementioned literature and our own observations, we have therefore hypothesized that this syndrome may serve as a "suicide trigger state" (ST state) mediating the transition to active suicide attempt in the potentially suicidal patient. Thus, identification of the proposed ST state in a high-risk population may be a powerful tool for the prediction of acute suicide risk.

Analysis of our data is suggestive of a state is marked by "ruminative flooding" (a confusing, uncontrollable and overwhelming profusion of negative thoughts) coupled with an acute, "frantic hopelessness", in which not only is there a fatalistic conviction that life cannot improve, but also an oppressive sense of entrapment and imminent doom. This builds to an intolerable, confused state in which patients feel that suicidal action is the only conceivable route of escape. In this state of severe distress, many patients have also reported the experience of "near-psychotic somatization" characterized by a concrete/somatic experience of thought, (e.g., thoughts creating head pressure) as well as somatic distortions (e.g., a subjective experience of a change in bodily size or shape).

In order to characterize the proposed ST state we have developed the Suicide Trigger Scale (STS), a rating scale that contains items testing for the above symptoms. Importantly, the STS does not rely on self-report of suicidal ideation. In this pilot study we aim to test the reliability and construct validity of the ST state as assessed by the STS-2, using statistical analysis of its coherence, internal structure, and relationship to a known validated instrument (the SCL-R 90). Further, we will assess the STS-2's relation to suicidal risk by examining the associations of scores on the scale and its individual components with a reported history of suicide attempt among patients with suicidal ideation.

## Methods

### Participants

The study was approved by the Beth Israel Institutional Review Board. Inclusion criteria were admission to psychiatric inpatient unit, chief complaint of suicidal wish/ideation upon admission, age ≥ 18 years, ability to understand and answer instrument questions, and literacy in the English language. The exclusion criteria were substance abuse in the 6 months prior to current hospitalization and a diagnosis of mental retardation or dementia. No other psychiatric diagnoses were exclusion criteria.

Subjects were recruited from the population of psychiatric patients receiving treatment at Beth Israel Medical Center's two non-dual diagnosis inpatient psychiatric units during the period of September 2006 through July 2008. During this time, of 2230 psychiatric admissions, a total of 141 (6.3%) met inclusion criteria, agreed to participate, signed the informed consent forms and provided sufficient data to be used in the study. Of these 130 (92.2%) completed all items on the STS-2 and 104 (73.8%) also completed the SCL-90R. Suicide attempt history was considered definitive if it was confirmed by participants' clinicians' consensus recorded in the chart at the time of their discharge. Suicide attempt history is obtained by policy as part of the admission assessment for all psychiatric inpatients at Beth Israel Medical Center. Due to administrative issues unrelated to this project, only 41 charts were available for the retrospective review of suicidal ideation and behavior.

Demographic and clinical data are presented in Table 1. Axis I diagnosis was unavailable for 15 subjects due to unavailability of their charts for review. The demographic characteristics of our population are comparable to those of large clinical trials such as the STAR*D,[28,29] demonstrating similar proportions of males and females and similar distributions of age and level of education, though in our sample a substantially higher percentage was identified as Hispanic while a lower percentage was identified as Caucasian. This difference reflects the demographics of the local population at large [30].

**Table 1:** Demographic and Clinical Variables

	All subjects (total N = 141)	PCA subjects (total N = 130)
***Means and standard deviations of dimensional demographic variables***		

	***Mean (SD)***	***Mean (SD)***

Age (range: 18-83)	42.2(*14.3 *)	42.4 *(14.4)*

Years of education (range: 4-20)	12.8 *(1.7)*	12.7 *(1.7)*

***Frequencies and percentages of categorical demographic variables***		

	***N(%)***	***N(%)***

Sex		
Female	85 *(60)*	77*(59)*
Male	56 *(40)*	53*(41)*

Relationship status (2 subjects missing data)		
***Total w/o partner***	***110 (78)***	***103(80)***
Single	84 (60)	79 (61)
Divorced	16 (11)	14 (11)
Widowed	4 (3)	4 (3)
Separated	6 (4)	6 (5)
***Total w/Partner***	***29 (21)***	***25 (19)***
In committed relationship	11 (8)	11 (8)
Married	18 (13)	14 (11)

Race		
Caucasian	69 (49)	63 (48)
Hispanic	48 (34)	44 (34)
Afro-American	14 (10)	14 (11)
Other/missing	6 (4)	5 (4)
Asian	4 (3)	4 (3)

Axis I diagnosis (15 subjects missing data)		
***Total MDD***	***43 (30)***	***41 (31)***
MDD	30 (21)	29 (22)
MDD with panic attacks	13 (9)	12 (9)
***Total bipolar***	***31 (21)***	***27 (20)***
Bipolar manic	19 (13)	16 (12)
Bipolar depressed	4 (3)	4 (3)
Bipolar mixed	4 (3)	4 (3)
Bipolar with panic attacks	3 (2)	3 (2)
***Total psychotic***	***29 (21)***	***27 (20)***
Schizoaffective/Schizophrenia	21 (15)	20 (15)
Psychosis NOS	8 (6)	7 (5)
***Total anxiety***	**25 (18)**	**21 (16)**
GAD with Panic Attacks	24 (17)	20 (15)
**Any diagnosis with panic attacks**	***40 (28)***	***35 (27)***

History of suicide attempt (SA)	12 (8.5)	11 (8.5)
History of SA denied	25 (17.7)	25 (19.2)
History of SA unknown	105 (74.5)	95 (73.1)

### Procedure and Instruments

The participants were approached by research assistants who explained the purpose of the study, the nature of the scales, the measures taken to ensure confidentiality of the disclosed information and subjects' right to refuse or stop participation. After signing informed consent forms, subjects were given the self-administered STS-2 and SCL-90R to complete. The scales were administered in no particular order. Research volunteers collected demographic information from patient charts after the questionnaires were completed. Diagnoses and medication information were obtained from the medical charts of the psychiatric unit.

#### Suicide Trigger Scale version 2 (STS-2)

The STS-2 (additional file 1) is a 39 item scale with 3 response categories (0 = not at all, 1 = somewhat, 2 = a lot) and is derived from STS-1 [31]. The STS-1 was originally given to 36 subjects on the same acute psychiatric units as STS-2 and re-administered 7-14 days later to those 13 who were still hospitalized (Cronbach's alpha 0.86;test re-test reliability 0.911)[31]. The scores had normal distribution. Exploratory factor analysis with the STS-1 revealed 4 factors with eigenvalues greater than 1. These were labeled Dread and Doom (Factor 1), Changes in Body (Factor 2), Head Pressure (Factor 3), and Hopelessness (Factor 4). After a consensus development meeting, the STS-1 was then revised by removing non-contributory items and adding new clinically-derived items to capture more symptoms of dissociation, somatization, head pain, and dread. The result was the 39-item STS-2.

#### The Symptom Checklist -90-Revised (SCL-90-R)

The SCL-90-R is a well-established 90-item scale with 5 response categories (0 = 'not at all' to 4 = 'very much') that assesses the presence and intensity of a wide variety of psychological symptoms [32]. The total score and 9 sub-scales were used in the analyses. The sub-scales of the SCL-90-R are Anxiety, Depression, Obsessive-Compulsive, Interpersonal Sensitivity, Somatization, Phobic Anxiety, Psychoticism, Hostility, and Paranoid Ideation, and have all been found to have high reliability with Cronbach's alphas ranging from 0.8 to 0.9, one-week test-retest reliability ranging from 0.8 to 0.9, and convergent validity with the Minnesota Multiphasic Personality Inventory (MMPI) [32]. Item 59, which assesses the presence of "thoughts of death," was also used in the analysis.

### Statistical Analysis

Reliability was assessed through Cronbach's alpha, which was used as a measure of internal consistency. Construct validity was assessed through a variety of statistical methods, including principal component analysis to explore the internal structure of the STS, Receiver Operator Characteristic (ROC) analysis with Fisher's exact test for cut-score to demonstrate clinical significance, and logistic regression analysis to examine which items of the STS-2 appeared to be most associated with suicidal action. Additionally, concurrent validity was assessed with correlation coefficients between STS-2 and SCL-90R scores and sub-scores.

### Internal Structure of the STS-2

Principal components analysis (PCA) with component rotation was used to assess the internal structure of the STS[33]. Because PCA requires pairwise-complete observations to calculate the correlation matrix that determines the factor loadings only data from those subjects (N = 130) who completed every item of the STS-2 could be used. (See Table 1 for comparison of PCA subjects and the total sample.) Three methods were used in succession to decide the number of components to be extracted in PCA: on first pass, eigenvalues >1, on second pass Scree plot, and finally, interpretability of components was used to eliminate components marginal on scree plot.

Following PCA, component rotation was performed by both Varimax rotation and Promax rotation, both with Kaiser Normalization. Varimax rotation preserves orthogonality of components while maximizing the variance of factor loadings on each component. The aim of this technique is to produce conceptually coherent, maximally independent, component subscales. Promax rotation does not preserve orthogonality, but aims to maximize component coherence and thus their semantic interpretability.

### Clinical Significance of the STS-2 - Construct Validity

Clinical significance of the STS-2 was assessed using ROC analysis of the STS-2 scores in discriminating past suicide attempters from those who had not made any suicide attempts[34]. ROC was performed on the unscaled STS to determine both Area Under the Curve (AUC) as a measure of the scale's robustness, and an optimal cut-score, the statistical significance of which was measured using Fisher's exact test. As the distributions of STS-2 scores in the PCA group and the subgroup chart-reviewed for suicide attempt history were very close (mean(standard deviation); 38(18) vs. 42(15), respectively), ROC analysis was also performed on the principal components produced in the Varimax PCA analysis to measure their robustness as discriminators between suicide attempters and non-attempters.

In addition, logistic regression analysis[35] was used to assess which individual items appeared to be most strongly associated with suicidality. Logistic regression analysis was used to produce a coefficient for each item of the STS-2 based on a separate regression of SA onto it. The resulting odds ratio is interpreted as the change in log-odds of SA when that item score increases by one.

### Concurrent Validity

Finally, scores on the STS-2 and its principal components were correlated with total and subscale scores on the SCL-90R as a measure of concurrent validity. Bonferroni correction for multiple (n = 30) comparisons was used to correct the threshold for statistical significance.

## Results

The scale showed a normal distribution of scores (p-values of the Shapiro-Wilk test of normality were 0.974 and 0.18 for the SA and non-SA groups respectively). For the 130 subjects who completed the STS-2, there was a mean score of 34 and standard deviation of 16.

### Reliability

The STS-2 showed high internal consistency with a Cronbach's alpha of 0.949. Four items (#13 trouble falling asleep, #16 panic attack, #29 ideas turning over and over, and #30 feeling doomed) were demonstrated to decrease Cronbach's alpha. Of these only one, 'doom', loaded strongly on our final principal component solution (see Table 2).

**Table 2:** Two-component solution: Promax rotation with Kaiser normalization

STS-2 numbered items	Component 1 factor loadings	Component 2 factor loadings
18. Strange sensations in body or on skin	.872	

19. Something happening to body	.847	

39. Headache from too many thoughts in head	.808	

5. Unusual physical sensations	.797	

20. Thoughts racing	.779	

21. Have no control	.743	

37. Pressure in head from thinking too much	.731	

6. Head could explode from too many thoughts	.699	

11. Head or body parts changed in size or shape	.658	

30. Doomed		.741

1. Wake up tired and not refreshed		.739

32. Would like troubling thoughts to go away but they won't		.737

34. Hope of change (reversed)		.679

23. Think things will be normal again (reversed)		.676

### Internal Structure

**Figure 1:**
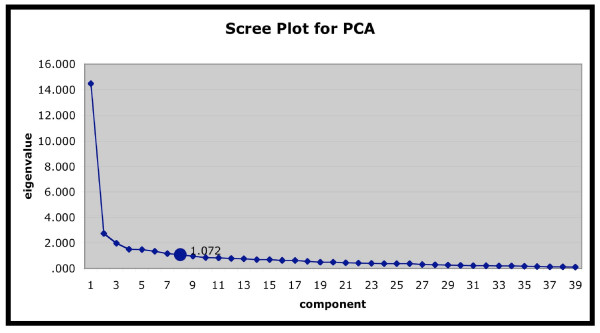
**Scree Plot for PCA**. The eigenvalue for each component generated by first-pass principal component analysis. Eight components had an eigenvalues >1.

Principal component analysis extracted 8 components with eigenvalues > 1, together accounting for 66% of the variance in the STS scores. The Scree plot suggests the use of one to three principal components (see Figure 1). However, the one-component solution lacked semantic coherence, while the three-component solution yielded two components approximately equivalent to the two-component solution followed by a minimally contributory and semantically incoherent third component. Thus the solution with two principal components accounting for 44% of the variance (37% and 7%, respectively), was found to best fit the data and was used as the basis for subsequent analysis.

Based on the two factor solution, we characterized the two principal components as follows:

Principal Component 1: Ruminative Flooding (thought experienced as a confusing and uncontrollable of flood of ruminative ideas) and Near-Psychotic Somatization (distorted/bizarre somatic perception and concrete/somatic experience of thought).

Principal Component 2: Frantic Hopelessness (acute, fatalistic conviction that one's situation is hopeless and life cannot improve compounded by a fearful and oppressive sense of entrapment and doom).

**Figure 2:**
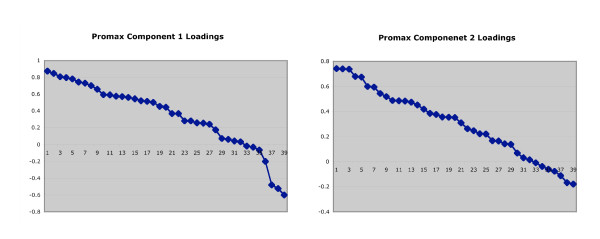
**Ordered factor loadings for the STS-2 individual items on principal components**.

The Varimax solution, which maintains component orthogonality, is very similar to the Promax solution presented here in Table 2. Inspection of the graphs of ordered factor loadings suggested an item loading cut-off value of 0.6 for both principal components (see Figure 2). The graphs show clusters of items loading similarly on a given factor, and inspection of items with similar loading values reveals generally similar content. Items describing a sense of entrapment (# 4,14,26,36) had substantial loadings (0.4-0.6) on both components but did not meet the cut-off threshold.

### Clinical significance - Construct Validity

**Figure 3:**
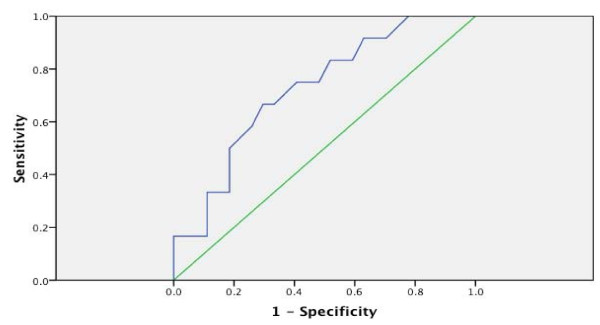
**The ROC curve for the global score on the STS-2**. The ROC Curve (blue) and reference line (green) for the STS-2 shows the sensitivity (probability of a true positive being detected) versus 1-specificity (probability of false positive) for the scale in identifying subjects with history of SA using incrementally decreased cut-off scores. Diagonal segments are produced by ties. The point of greatest separation between the ROC curve and the reference line marks the sensitivity (.667) and specificity (.774) of the optimal cut-off score.

ROC analysis of the STS-2 raw scores (N = 36) showed significant and robust detection of a reported history of suicide among suicidal ideators with an AUC of 0.724 and asymptotic significance of 0.027. Analysis of the ROC curve suggests an optimal cut-score of 48 (approximately one standard deviation above the sample mean). Sensitivity for a cut-off total STS-2 score of 48 is 0.667, specificity is 0.704 and the 1-sided p-value of the Fisher exact test is significant at the 0.02 level (see Figure 3).

### ROC analysis of subscales

ROC analysis of both Promax and Varimax 2-component solutions found statistically significant (asymptotic p = 0.002) prediction of suicide attempt history in the second component, (Frantic Hopelessness) with AUCs of 0.83 and 0.82, respectively. This finding correlates well with the results of the logistic regression on the individual items discussed below.

### Regression analysis

Logistic regression was performed to determine the association between each STS-2 item and the reported history of suicide attempt (N = 36). Regression coefficients and uncorrected p-values for STS-2 individual items regressed onto reported history of SA are presented in Table 3. Although logistic regression analysis of the individual items of the STS-2 against history of SA found no statistically significant results after Bonferroni correction for multiple comparisons (required p value <0.00128), this criterion may be excessively stringent [36]. The items with the highest coefficients were all descriptive of one of three themes: ruminative flooding, doom/hopelessness, and entrapment. Item #33 (can stop thoughts that are troubling) had the highest odds ratio (16.01). In other words, subjects who endorsed a score of 2 ("a lot") were approximately 16 times more likely to have had a previous suicide attempt than subjects who endorsed a score of 1 ("somewhat"). Likewise, 9 items describing ruminative flooding (Items #2, 3, 9, 12, 13, 20, 29, 32, and 33) had a mean regression coefficient of 0.97 (corresponding to an OR of 2.64). Contrary to expectations, items describing near-psychotic somatization (Items #5, 11, 18, 19 and 24) produced negative coefficients in the regression analysis (albeit only at an uncorrected trend level of significance). Thus in our sample population of psychiatric inpatients, more bizarre somatic experience corresponded to a decreased likelihood of having made a past suicide attempt.

**Table 3:** Regression coefficients and uncorrected p-values for STS-2 individual items regressed onto reported history of SA

STS-2 numbered items	Regression coefficient	p-value
33. Can stop thoughts that are troubling (reverse scored)	2.77	0.01

4. No exit	2.42	0.03

30. Doomed	2.02	0.01

36. No escape	1.96	0.02

28. Sense of dread	1.76	0.02

38. Think you will ever feel better (reverse)	1.69	0.03

9. Hard to stop worrying	1.57	0.01

13. Trouble falling asleep because of thoughts you cannot control	1.54	0.02

17. Expect the worst	1.49	0.07

34. Hope of change (reverse)	1.45	0.01

23. Think things will be normal again (reverse)	1.42	0.01

26. Trapped	1.39	0.02

35. Something horrible going to happen	1.20	0.05

12. Cannot concentrate or make decisions due to too many thoughts	1.05	0.05

32. Would like troubling thoughts to go away but they won't	1.05	0.07

16. Sudden panic-attack or physical symptoms	0.94	0.12

15. World feels different	0.71	0.14

1. Wake up tired and not refreshed	0.68	0.17

27. Feel blood rushing through veins	0.66	0.17

25. Helpless to change	0.64	0.23

14. World closing in	0.59	0.26

7. Ordinary things look strange or distorted	0.56	0.33

29. Ideas turning over and over, won't go away	0.55	0.35

10. Hopeless	0.41	0.43

20. Thoughts racing	0.33	0.50

6. Head could explode from too many thoughts	0.29	0.56

21. Have no control	0.19	0.69

8. Worry bad things might happen	0.19	0.72

2. Thoughts confused	-0.02	0.98

3. Many thoughts in head	-0.10	0.89

22. Bothered by thoughts that do not make sense	-0.11	0.82

31. Something wrong physically	-0.11	0.81

39. Headache from too many thoughts in head	-0.24	0.59

5. Unusual physical sensations	-0.46	0.32

19. Something happening to body	-0.62	0.15

37. Pressure in head from thinking too much	-0.87	0.11

18. Strange sensations in body or on skin	-0.92	0.07

24. Sensations you cannot describe	-1.14	0.06

11. Head or body parts changed in size or shape	-1.41	0.06

### Integration of Principal Component and Regression Analyses

Several of the best-performing items in regression analysis loaded strongly (factor loading values ≥ 0.5) on the principal components. Furthermore, items with relatively high regression coefficients (> 1.0) had a strong mean loading of 0.46 on Principal Component 2 (which was a robust detector of past SA), but a weak mean loading (0.15) on Principal Component 1 (which performed poorly as a detector of past SA under ROC analysis). In combination with the heavy loading of somatic symptoms on Component 1, this appears to account for Component 1's poor performance as a predictor of suicide attempt history on ROC analysis.

### Concurrent and External validity of the STS-2

One hundred and four (104) subjects completed both the SCL-90-R and the STS-2. Correlations between STS-2 total score and principal component 1 and 2 scores were calculated and correlated with the SCL-90-R total scores, the nine subscales and Item 59 - "Thoughts of death or dying". There was a high correlation between total scores on the STS-2 and the SCL-90; r = 0.77. High correlations were found for all subscales, principally for depression and anxiety. The lowest correlation coefficient was found for Item 59. However this is most likely an artifact of the low range of scores possible for a single item as compared to a subscale, which makes it more susceptible to noise. The results are shown in Table 4 below. All correlations were significant to p < 0.001, (equivalent to p < 0.03 after Bonferroni correction for multiple comparisons).

**Table 4:** Correlation coefficients (r) between STS-2 scores and SCL-90 sub-scale scores

	STS-2 total score	Principal comp. 1 score	Principal comp. 2 score
**SCL5- Anxiety**	0.79	0.80	0.79

**SCL4- Depression**	0.71	0.75	0.72

**SCL2- Obsessive Compulsive**	0.69	0.70	0.71

**SCL3-Interpersonal Sensitivity**	0.67	0.67	0.68

**SCL1- Somatization**	0.63	0.64	0.65

**SCL7- Phobic Anxiety**	0.62	0.62	0.63

**SCL9 -Psychoticism**	0.61	0.61	0.63

**SCL6- Hostility**	0.56	0.55	0.57

**SCL8- Paranoid Ideation**	0.53	0.51	0.55

**Item 59- Thoughts of death**	0.53	0.58	0.55

Substantial numbers of high STS-2 scores were found in all demographic and diagnostic subgroups, demonstrating that the instrument measures a state that is not demographically bound, and is distinct from panic, mood, and psychotic disorder. Table 5 shows the mean scores on the STS-2 across demographic and diagnostic variables as well as the percentage and N of each demographic subgroup of the entire sample that scored above the cut-score. While substantial differences may be noted between different demographic subgroups, a substantial proportion (> 20%) of each subgroup reported a score greater than the cut-score. Comparison of demographic and diagnostic categories by Fisher exact test demonstrated no significant differences at the p < 0.05 level, providing preliminary evidence of external and divergent validity.

**Table 5:** STS-2 Scores by demographic subgroup

Demographic	STS score: Mean (SD)	N(%) with score > 48
Sex		
Female	39.8 (17.6)	32 (38)
Male	36.4 (17.6)	16 (29)

Race		
Caucasian	36.1 (14.75)	21 (30)
Hispanic	34.8 (18.6)	20 (42)
African-American	29.1 (13.8)	3 (21)

Primary Axis I diagnosis		
*MDD*	36.1 (15.0)	12 (28)
Bipolar	32.1 (17.6)	9 (29)
Psychotic	32.6 (15.2)	9 (31)
Anxiety D/O with panic attacks	35.5 (17.2)	11 (45)

Total With Panic Dx in Axis I	38.6 (16.1)	17 (44)
Total Without Panic Dx in Axis I	32.2 (15.8)	23 (23)

History of SA	44.45 (11.1)	8 (67)
No History of SA	36.4 (14.2)	8 (32)

## Discussion

The results of this preliminary investigation are limited by its retrospective design, reliance on self-report, relatively small size of the whole sample and of an even smaller subgroup of subjects with data on past suicide attempts. Thus, our findings should be viewed as exploratory in nature and are not intended to demonstrate causality or define a definitive component structure. Nonetheless, the high Cronbach's alpha suggests that the STS-2 defines a coherent psychopathological clinical state, and principal component analysis, though underpowered by a factor of two, is suggestive of two principal components.

The first component was termed Ruminative Flooding and Near-Psychotic Somatization, while the second was termed Frantic Hopelessness. Items describing entrapment and dread loaded strongly though below the cut-off level for both components, and were found in regression analysis to be highly sensitive to past SA. We conceptualize entrapment and dread as elements of Frantic Hopelessness. High scores on the STS-2 demonstrated significant sensitivity and specificity in distinguishing suicidal ideators with a history of attempt from those without. Finally there were high correlations between scores on the STS-2 and the SCL-90-R assessment of general psychopathology, as well as the depression and anxiety subscales of the SCL90-R, consistent with the conception of the suicide trigger state as a syndrome of disordered thought and affect. Our findings appear to be the first quantitative description of a discrete psychopathologic state other than suicidal ideation, and distinct from Axis I diagnosis, that demonstrates a differential association with suicidal action.

Our data supports our hypothesis that this state is associated with suicidal action, but cannot demonstrate causality. Further investigation is warranted to determine whether this state indeed serves as an acute trigger state for suicidal actions or, alternatively, serves as a marker of a trait susceptibility to taking suicidal action. Our results indicate that items encoding Ruminative Flooding and Frantic Hopelessness, including those describing entrapment and dread, were particularly associated with history of suicide attempt and thus may play a more prominent mediating role in the precipitation of suicidal action.

Combining the results from all our statistical analyses, our data paint a picture of a panic-like state characterized by disturbed thought process (rumination, perceptual distortion, near-psychotic somatization), and a pathological cathexis of thought content and affective arousal which we term 'frantic hopelessness.' In this state, hopelessness is acutely sharpened to a sense of doom, entrapment and dread.

The robustness of the second principal component of the STS-2 (Frantic Hopelessness) in distinguishing ideators with history of attempt from those without is consistent with the literature that identifies hopelessness as a primary risk factor for suicide attempt[37]-[38-40]. It might be argued that indeed our results no more than recapitulate Beck's finding that hopelessness is a strong predictor of suicidality. We suggest however that the coherence of the STS-2 demonstrated by its high Cronbach's alpha combined with the scale's inclusion of many items which are clearly distinct from hopelessness on face value, argues for a unique clinical syndrome broader in scope than hopelessness alone as described by Beck. Furthermore, the second principal component, while including elements akin to canonically described hopelessness, is distinct not only by virtue of existing within the context of this syndrome, but also because it contains items - such as doom (#30), fatigue (#1), and cognitive oppression (#32) - which lend it an acute, fatalistic and oppressive quality not previously described. This finding however is limited by lack of power for a definitive factor analysis.

Though Cronbach's alpha was high, two items, doom (#30) and panic attacks (#16) reduced this metric. That Cronbach's alpha was decreased by item 30 "Doom" could suggest that doom does not belong to the syndrome. However, Cronbach's alpha was not decreased by semantically similar items, or by other items that loaded most heavily on the Frantic Hopelessness component. An alternative explanation may be that 'doom', a somewhat literary word, was not familiar in the vocabulary of some subjects, and perhaps more so given the high proportion of Hispanic subjects, many of whom may not have been raised in an English-speaking environment. Similarly, item 16 "panic attack" may have reduced Cronbach's alpha because it relies upon subject familiarity or comfort with this technical term, which may not be as common in the lay vocabulary as, for example, "depression." Further, the high correlation of the total STS-2 scores and the two principal components with the SCL90-R Anxiety Subscale is consistent with the literature supporting panic and anxiety disorders as risk factors for suicide attempt [23,41,42,4].

Our finding that those items in the first principal component which are descriptive of Ruminative Flooding (such as racing and too many thoughts) generally produced fairly high regression coefficients (mean value 0.97) is consistent with the findings of Morrison and O'Connor[19,43] who identify ruminative thought as a suicide risk factor. The high correlation between STS-2 and SCL-90R total scores is in agreement with the literature that finds general severity of psychopathology to be a risk factor for suicide[4,44,45].

The marked variability of SCL-90R Item 59 (thoughts of death or dying) in a sample population of patients presenting with SI highlights the limited reliability of patient self report of SI. The comparatively low correlation between scores on item 59, which should, a priori, be high for suicidal ideators, and scores on the STS-2 items most predictive of past SA as grouped in Component 2, highlights the importance of a clinical measure which does not rely on overt self-report of suicidality.

Our results also present the unexpected finding that items of the STS-2 that describe near-psychotic somatization (which could be interpreted as variants of somatic and dissociative symptoms of panic attack) appear to correlate negatively - though not significantly - with history of SA. This is contrary to the literature linking suicide risk to panic attacks, and overall severity of psychopathology and psychoticism[21,24,45]. While our data are not sufficiently powered to demonstrate this, inspection of score distributions across different axis I diagnoses suggests that schizoaffective subjects were more heavily represented among those with history of SA but had lower scores on the STS-2 somatization items, while subjects scoring highest on somatization items were rather those with combined depression and anxiety diagnoses. Possibly this is merely an artifact of small sample size and sample population. We speculate however, that among those subjects with primary anxiety diagnoses, somatization is a marker of concern for bodily integrity (as in the hypochondriac) and may protect against self-harm behaviors [46,47].

As highlighted, our study has a number of limitations. In summary, while the study has the advantage of comprising a demographically and diagnostically balanced population, it is limited in sample size and was not sufficiently powered to reliably detect differences between subgroups. Furthermore, the sample size is too small for a definitive factor analytic study and thus the factor structure should be considered preliminary. The limitations imposed on the secondary analyses by small sample size were magnified by the lack of availability of complete clinical data for many subjects due to lack of chart availability, such that Axis I diagnosis unknown for 15 subjects and suicide attempt history was only known for 39 subjects. Though there were no significant differences between the subject group as a whole and the subgroup of subjects whose charts were available for review of SA history in terms of ethnic group composition, or scores on the STS-2, a significantly higher proportion of the entire group carried bipolar and psychotic disorder diagnoses than in the chart-reviewed subgroup (approximately 40% vs. 25%, p = 0.04). The cultural diversity of the sample may also affect the results in ways which the current study is unable to account for due to cultural mediation of symptomatology; somatic symptoms in particular may exhibit culturally mediated differences in salience, semantic significance, and prognostic value [48,49]. A further limitation common to studies of infrequent phenomena such as suicide is its retrospective design, and, in particular, its reliance on self-report as the only measure of suicide attempt history. As with all self-report instruments, there is risk that subjects did not understand all of the scale items, answer accurately, or without bias.

## Conclusions

Within the study limitations, our findings suggest that the STS-2 describes a novel and coherent syndrome of psychic experience, separate from suicidal ideation and DSM-IV axis I diagnosis, which demonstrates an association with report of past suicidal action. This state consists of ruminative flooding, near-psychotic somatization and frantic hopelessness. Scores on the STS-2 can distinguish between suicidal ideators who report having made an attempt in the past from those who deny past suicide attempts.

There is a great need for a reliable and valid instrument that would enable health care professionals to identify patients at increased risk of acting on their ideations and to pre-empt serious suicide attempts, particularly in those patients at greater risk for "low plan" or impulsive suicide or those who deliberately conceal or unconsciously repress suicidal ideation[14,15]. Thus, an assessment that does not rely heavily on the self-reported cognitions of patients would be of particular value. The lack of emphasis on suicidal ideation and plan in the STS-2 could make it particularly suited to this task, as these features may be absent, outside of conscious awareness, or may be intentionally underreported. Future larger studies utilizing prospective approaches, larger samples, and corroborated suicidal events are therefore needed to substantiate the current results and establish the STS-2 as a predictor of suicidal action. Future studies should also explore the influence of culture, gender, and primary psychiatric diagnosis on STS global scores and subscales, to demonstrate its ability to predict suicide acutely and prospectively and to further elucidate which elements of the state are most predictive of suicide attempts.

## Competing interests

The authors declare that they have no competing interests.

## Authors' contributions

ZY drafted the manuscript and contributed the design and completion of the data analyses. CK assisted in the drafting of the manuscript, performance of the statistical analyses, as well as the coordination of the study. MSJ designed and performed the principal statistical analyses. DE and LJC provided substantial editorial input in the drafting of the manuscript. IIG conceived of the study, and participated in its design and coordination and helped to draft the manuscript. All authors read and approved the final manuscript.

## Pre-publication history

The pre-publication history for this paper can be accessed here:

http://www.biomedcentral.com/1471-244X/10/110/prepub

## Supplementary Material

Additional file 1 **STS-2 PDF**. Click here for file
