# Supplementary material for: Construct development: The Suicide Trigger Scale (STS-2), a measure of a hypothesized suicide trigger state
Source: BMC Psychiatry. 2010 Dec 14;10:110. doi: 10.1186/1471-244X-10-110 (PMC3016314; doi:10.1186/1471-244X-10-110)
Supplement: Additional file 1 — STS-2 PDF. [file 1471-244X-10-110-S1.PDF]

STS version 2 -- 39 items;

Date:
Assessment #:
Subject ID#:
Rater:

There may have been times in your life when you felt particularly anxious and may have been having panic attacks. I am going to ask you questions about how you may have felt during the time when you had the most severe anxiety or panic attacks, In some people those happen when life gets very difficult or depressing. During those times,

1. When you get very anxious, do you wake up from sleep feeling tired and not refreshed?
   0= Not at all
   1= Somewhat
   2= A lot

2. When you get very anxious, do you feel your thoughts are confused?
   0= Not at all
   1= Somewhat
   2= A lot

3. When you get very anxious, do you have many thoughts in your head?
   0= Not at all
   1= Somewhat
   2= A lot

4. When you get very anxious, do you feel there is no exit?
   0= Not at all
   1= Somewhat
   2= A lot

5. When you get very anxious, do you feel unusual physical sensations that you have never felt before?
   0= Not at all
   1= Somewhat
   2= A lot

6. When you get very anxious, do you feel that your head could explode from too many thoughts?
   0= Not at all
   1= Somewhat
   2= A lot

7. When you get very anxious, do you have the feeling that ordinary things look strange or distorted to you?

0= Not at all
1= Somewhat
2= A lot

8. When you get very anxious, do you ever worry that a lot of bad things might happen to you?

0= Not at all
1= Somewhat
2= A lot

9. When you get very anxious, do you feel that it is hard for you stop worrying?

0= Not at all
1= Somewhat
2= A lot

10. When you get very anxious, do you feel hopeless?

0= Not at all
1= Somewhat
2= A lot

11. When you get very anxious, do you feel that your head or body parts have changed in size or shape?

0= Not at all
1= Somewhat
2= A lot

12. When you get very anxious, do you have a decreased ability to think, concentrate or make decisions, due to too many thoughts?

0= Not at all
1= Somewhat
2= A lot

13. When you get very anxious, do you have trouble falling asleep from because you are having thoughts you cannot control?

0= Not at all
1= Somewhat
2= A lot

14. When you get very anxious, do you feel that the world is closing in on you?

0= Not at all
1= Somewhat
2= A lot

15. When you get very anxious, does the world around you feel different?
0= Not at all
1= Somewhat
2= A lot

16. When you get very anxious, do you ever feel suddenly frightened to such an extent that you develop physical symptoms or have a panic attack?
0= Not at all
1= Somewhat
2= A lot

17. When you get very anxious, do you expect the worst?
0= Not at all
1= Somewhat
2= A lot

18. When you get very anxious, do you have strange sensations in your body or on your skin?
0= Not at all
1= Somewhat
2= A lot

19. When you get very anxious, do you feel that something is happening to a part or parts of your body?
0= Not at all
1= Somewhat
2= A lot

20. When you get very anxious, do you feel your thoughts are racing?
0= Not at all
1= Somewhat
2= A lot

21. When you get very anxious, do you feel you have no control?
0= Not at all
1= Somewhat
2= A lot

22. When you get very anxious, are you bothered by thoughts that did not make sense?
0= Not at all
1= Somewhat
2= A lot

23. When you get very anxious, do you feel things will ever be normal again?
0= Not at all
1= Somewhat

2= A lot

24. When you get very anxious, do you have sensations that you can not describe?
0= Not at all
1= Somewhat
2= A lot

25. When you get very anxious, do you feel helpless to change?
0= Not at all
1= Somewhat
2= A lot

26. When you get very anxious, do you feel trapped?
0= Not at all
1= Somewhat
2= A lot

27. When you get very anxious, do you feel blood rushing through your veins?
0= Not at all
1= Somewhat
2= A lot

28. When you get very anxious, do you feel a sense of dread?
0= Not at all
1= Somewhat
2= A lot

29. When you get very anxious, do you feel that ideas keep turning over and over in your mind and they won't go away?
0= Not at all
1= Somewhat
2= A lot

30. When you get very anxious, do you feel doomed?
0= Not at all
1= Somewhat
2= A lot

31. When you get very anxious, do you feel something is wrong with you physically?
0= Not at all
1= Somewhat
2= A lot

32. When you get very anxious, would you like your troubling thoughts to go away but they won't?
0= Not at all

1= Somewhat
2= A lot

33. When you get very anxious, can you stop thoughts that are troubling you?
0= Not at all
1= Somewhat
2= A lot

34. When you get very anxious, is there hope that things are going to change?
0= Not at all
1= Somewhat
2= A lot

35. When you get very anxious, do you feel like something horrible is going to happen?
0= Not at all
1= Somewhat
2= A lot

36. When you get very anxious, do you feel there is no escape?
0= Not at all
1= Somewhat
2= A lot

37. When you get very anxious, do you feel pressure in your head from thinking too much?
0= Not at all
1= Somewhat
2= A lot

38. When you get very anxious, do you think you will ever feel better?
0= Not at all
1= Somewhat
2= A lot

39. When you get very anxious, do you get a headache from too many thoughts in your head?
0= Not at all
1= Somewhat
2= A lot
